# Efficient Universal Computing Architectures for Decoding Neural Activity

**DOI:** 10.1371/journal.pone.0042492

**Published:** 2012-09-12

**Authors:** Benjamin I. Rapoport, Lorenzo Turicchia, Woradorn Wattanapanitch, Thomas J. Davidson, Rahul Sarpeshkar

**Affiliations:** 1 M.D.–Ph.D. Program, Harvard Medical School, Boston, Massachusetts, United States of America; 2 Department of Electrical Engineering and Computer Science, Massachusetts Institute of Technology, Cambridge, Massachusetts, United States of America; 3 Division of Health Sciences and Technology, Harvard University and Massachusetts Institute of Technology, Cambridge, Massachusetts, United States of America; 4 Department of Bioengineering, Stanford University School of Medicine, Stanford, California, United States of America; University of Michigan, United States of America

## Abstract

The ability to decode neural activity into meaningful control signals for prosthetic devices is critical to the development of clinically useful brain– machine interfaces (BMIs). Such systems require input from tens to hundreds of brain-implanted recording electrodes in order to deliver robust and accurate performance; in serving that primary function they should also minimize power dissipation in order to avoid damaging neural tissue; and they should transmit data wirelessly in order to minimize the risk of infection associated with chronic, transcutaneous implants. Electronic architectures for brain– machine interfaces must therefore minimize size and power consumption, while maximizing the ability to compress data to be transmitted over limited-bandwidth wireless channels. Here we present a system of extremely low computational complexity, designed for real-time decoding of neural signals, and suited for highly scalable implantable systems. Our programmable architecture is an explicit implementation of a universal computing machine emulating the dynamics of a network of integrate-and-fire neurons; it requires no arithmetic operations except for counting, and decodes neural signals using only computationally inexpensive logic operations. The simplicity of this architecture does not compromise its ability to compress raw neural data by factors greater than 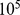. We describe a set of decoding algorithms based on this computational architecture, one designed to operate within an implanted system, minimizing its power consumption and data transmission bandwidth; and a complementary set of algorithms for learning, programming the decoder, and postprocessing the decoded output, designed to operate in an external, nonimplanted unit. The implementation of the implantable portion is estimated to require fewer than 5000 operations per second. A proof-of-concept, 32-channel field-programmable gate array (FPGA) implementation of this portion is consequently energy efficient. We validate the performance of our overall system by decoding electrophysiologic data from a behaving rodent.

## Introduction

### Implantable Neural Decoding Systems for Brain– Machine Interfaces

Recent years have seen dramatic progress in the field of brain– machine interfaces, with implications for rehabilitation medicine and basic neuroscience [1]–[3]. One emerging goal is the development of an implantable system capable of recording and decoding neural signals, and wirelessly transmitting raw and processed neural data to external devices. Early versions of such systems have shown promise in developing prosthetic devices for paralyzed patients [4], retinal implants to restore sight to the blind [5], [6], deep brain stimulators for treating Parkinson's disease and related disorders [7], and systems for predicting and preventing seizures [8]. Neural decoding has been essential to many of these systems, conferring the adaptive ability to learn to extract from neural data meaningful signals for controlling external devices in real time.

Electronics implanted in the brain must be sufficiently energy-efficient to dissipate very little power while operating, so as to avoid damaging neural tissue; conserving power also extends device lifetimes and reduces system size [9]. Yet experimental and clinical neuroscience demand ever-increasing bandwidth from such systems [10]: sampling rates on the order of 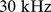 per recording channel are commonly used in applications requiring discrimination of action potentials generated by individual cells, and while contemporary systems rarely record from more than hundreds of neurons simultaneously, much more extensive sampling will be required to probe the state of an entire human brain containing on the order of 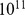 neurons. In this context, neural decoding can be viewed not only as a computational approach to extracting meaning from vast quantities of data [11], but also as a means of compressing such data. Neural decoding as a type of compression is ‘lossy’ in the formal sense that the decoding operation cannot be inverted to reproduce the original neural input signals, given only the decoder output. However, in neural prosthetics and related applications, not the neural signals but rather the information they encode is of primary interest. Indeed, the neural signals themselves can be viewed as constituting a redundant representation of underlying state information. In such contexts, the principal concern is not lost neurophysiologic information, but rather faithful reconstruction of encoded information, such as movement trajectories. The compression ratio, power consumption, and correlation of decoder output with encoded states and trajectories are relevant measures of performance. In previous work [12], [13], we have shown that an implanted neural decoder can compress neural data by a factor of 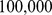.

Considerable attention has been devoted to meeting the low-power operation constraint for brain implantation in the context of signal amplification [14], [15], analog-to-digital conversion [16], power and data telemetry [17]–[20], neural stimulation [21]–[23], and overall low-power circuit and system architecture [9], [24]. A small amount of work has also been conducted on power-efficient neural data compression [25]. However, almost no systematic effort has been devoted to the problem of power-efficient neural decoding [12], [13].

**Figure 1:**
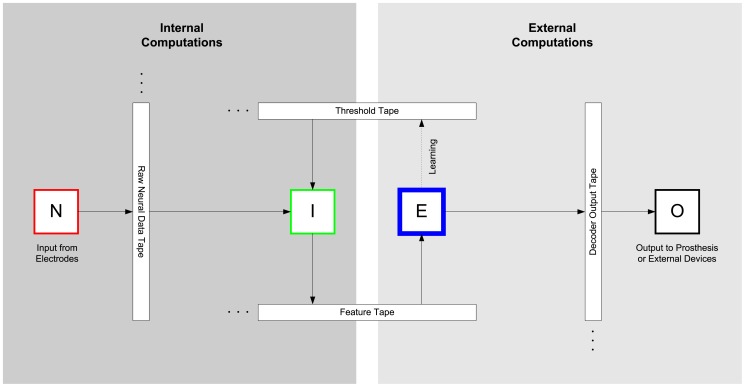
Universal Computing Architecture for Neural Decoding. The overall architecture of a neural decoding system is decomposed into a set of operations implemented by Turing-type computing machines, shown here as a collection of *heads* (data processing units) reading from and writing to a set of corresponding *tapes* (programs and data streams). Amplification and digitization of raw neural data, and decoding of that data, are performed by heads *N* and *I*, respectively, in a biologically implanted unit. The ‘Internal Computations’ of these two system components are streamed across a wireless data channel to an external unit, which performs more power-intensive ‘External Computations’ to post-process the decoded output. Further processing of the decoded data is performed externally by head *E*, and the final output of the system is reported by head *O*. The external system implements a learning algorithm that is used to write the program on the threshold tape, which is executed by the internal unit.

Multiple approaches to neural decoding have been implemented by several research groups. As we have discussed in [12], nearly all of these have employed highly programmable algorithms, using software or microprocessors located outside the body [26]–[41]. An implantable, low-power decoder, designed to complement and integrate with existing approaches, would add the efficiency of embedded preprocessing options to the flexibility of a general-purpose external processor. As illustrated in Figure 1, our decoding architecture is designed to couple a power-efficient, bandwidth-reducing, implanted decoder, with an external unit that is less power-constrained and can therefore bear a computational load of greater complexity when postprocessing the decoded neural data. Being optimized for low power consumption, it sacrifices a small amount of algorithmic programmability—posing algorithmic challenges with which we deal in this paper—to reduce power consumption and physical size, and to facilitate inclusion of the decoder within an implanted unit.

As we have described in previous work [9], [24], such an implanted unit consists of circuits for neural signal amplification, digitization, decoding, and near-field power and data telemetry. In association with an external unit that manages wireless power transfer and far-field data telemetry, such an implanted unit forms the electronic core of a brain– machine interface.

### Biological and Universal Computing Primitives for Neural Decoding

Our computational architecture for neural decoding operates explicitly as a Turing-type universal computing machine, in which the decoding operation is programmed by selecting the rule array of the machine, which can also reprogram itself, resulting in an overall system that emulates the dynamics of a network of integrate-and-fire neurons. In contrast with existing approaches to neural decoding, this framework facilitates extreme power efficiency, *requiring no arithmetic operations except for counting*.

Our architecture decomposes the operation of neural signal decoding, allocating the computational load across two processing units: one implanted within the body, and therefore power-constrained, and the other located outside the body, and therefore less power-constrained. The overall architecture strategically imbalances the computational load of decoding in a way that leverages the relatively high computational power of the external unit to minimize power consumption in the implanted unit. Simultaneously, the system minimizes data throughput between the internal and external units in order to reduce the power costs of wireless communication between the two units.

Figure 1 shows the overall architecture of our neural decoding system. The architecture is decomposed into a set of operations implemented by Turing-type computing machines, shown as a collection of heads (data processing units) reading from and writing to a set of corresponding tapes (programs and data streams). Amplification and digitization of raw neural data, and decoding of that data, are performed by heads *N* and *I*, respectively, in the implanted unit. The computations of these two system components are streamed across a wireless data channel to an external unit, which performs more power-intensive external computations to postprocess the decoded output. In particular, further processing of the decoded data is performed externally by head *E*, and the final output of the system is reported by head *O*.

The core decoding function executed by the internal unit is an evaluation of the probability that the system is in each of its available states. At each time step, 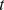, the internal unit reports a one-bit binary score 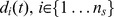 for each of the 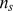 possible states, based on neural data observed at each time step. The binary vector of scores, 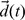, is processed by the external unit, which decides, on the basis of system history and *a priori* information, which single state is most probable. It then broadcasts its decision, for example to be used in controlling external devices.

**Figure 2:**
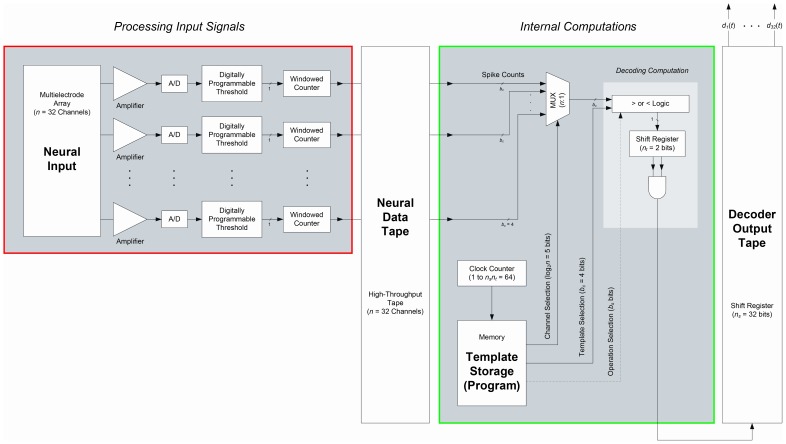
Decoding Architecture. Block diagram of the low-power processing system of the internal component of our neural decoder, as implemented in one instantiation of our architecture. Functional blocks are color-coded in accord with the scheme used in Figure 1.

The detailed operation of the internal unit is diagrammed in Figure 2, in which functional blocks are color-coded in accord with the scheme used in Figure 1. Neural inputs from an (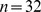)-channel array are amplified and digitized, and the resulting digital bits are copied to the high-throughput neural data tape. As indicated by the red rectangle in Figure 2, these operations correspond to the function of the *N* head in Figure 1. The internal decoding computations implemented by the *I* head in Figure 1 are shown in detail within the green box in Figure 2. Digital circuits in this subsystem monitor each input channel during successive time windows of length 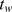, counting the number of spikes whose amplitudes exceed channel-specific, programmable levels. The resulting spike counts are evaluated by a program stored in memory, which constitutes the core of the internal decoder. The program defines a set of rules, one or more for each possible state, that are configured during a learning period and that are then used to evaluate the scores 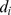 on the basis of the spike counts observed at each time step. Each rule also identifies the 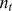 channels that are most informative in decoding its corresponding state, and typically only these channels are used for computing 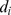 for state 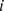. The spike-count thresholds for a given state-dependent rule are set through statistical learning such that the state may be discriminated from others with sensitivity and specificity that may be tuned to yield acceptable performance.

We use the terms *sensitivity*, *specificity*, and later *positive predictive value*, as they are classically used in the context of binary classification [42]. As we have cast the decoding problem, the decoder must implement a binary classification function at each time step, for each state, in deciding whether or not the observed neural firing pattern encodes that state; the results of these classifications are recorded as the components 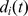. According to this framework, decoder sensitivity with respect to a given state, 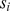, is defined as the proportion of cases in which state 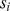 is correctly decoded by 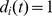; single-channel sensitivity is defined analogously, by restricting decoder input to a particular channel. Similarly, decoder specificity with respect to state 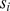 is defined as the proportion of cases in which states other than 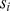, or the collective state 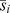 (*not*-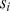), is correctly classified with respect to 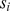 by 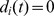; single-channel specificity is also defined analogously. The positive predictive value of decoding with respect to state 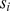 is defined as the proportion of positive, 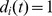, classification decisions that are correct; single-channel positive predictive value is again defined analogously. Finally, it is important to note that sensitivity and specificity are properties of the decoding function alone, independent of neural firing patterns, whereas positive predictive values depend on the distributions of recorded neural spikes [42].

The components of 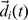 are written to the decoder output tape. The external unit examines the decoder output tape in a noncausal manner, postprocessing the decoded output generated by the internal unit to find a single most probable state. Our architecture permits a wide variety of postprocessing schemes, consistent with comparative studies of neural decoding algorithms and their underlying assumptions, which have formally and systematically demonstrated that movement smoothing is the most significant algorithmic factor influencing decoder performance [43]. We therefore implement a general-purpose decoding algorithm in the implanted system, while permitting application-specific choices in the external unit. Here we implement the postprocessing using a Viterbi algorithm.

Pattern matching algorithms conceptually related to the one implemented in our internal unit have previously been used to decode neuronal activity, notably in the context of memory replay during sleep [44], [45], but until now the computational complexity of such approaches has limited their applicability to off-line, software-based implementations. Pattern matching systems have the useful property of being able to emulate receptive field structures—essential computational primitives of biological neurons—in a direct and intuitive way: they learn and store a set of *templates*, patterns corresponding to the activity of a given ensemble of neurons in response to a particular set of external states. Classical implementations of decoding by pattern matching function by comparing observed neuronal activity against stored templates (the system must store at least one template for each state to be decoded) and choosing a best match. This approach is typically computationally expensive for two reasons. First, the ability to quantify the degree to which observed neuronal activity matches a given template requires a defined metric, the value of which must be computed for every stored template at every time step of the decoder. And second, useful metrics themselves typically require computationally expensive operations, such as multiplication, root extraction, and division (or normalization). Computation of continuous-valued metrics in a digital context can also be accomplished only to a specified limit of precision.

The efficiency of application-specific digital microcontrollers and digital signal processors (DSPs) arises in large part from their ability to identify and prioritize the computational primitives, such as Fourier transformation or specific kinds of filtering, that are of greatest importance in particular applications [46]. In seeking a minimal digital decoding system whose operation is consistent with the computing primitives of biological neural networks, we have retained pattern matching as an approach to embedding neuronal receptive fields within the decoding architecture. However, we have reduced the template-matching metric to a set of rules in programmable logic. The structure of these rules as implemented in the example system described here results in a decoding architecture that behaves like a network of integrate-and-fire neurons. However, the programmability of the system and its explicitly rule-based architecture ensure that its scope encompasses even complex, multimodal receptive fields, but is not limited to such emulations [47]; the decoding architecture presented here is an example of a universal computing machine customized for neural decoding.

This paper is structured as follows: In this Introduction Section and in the Discussion Section, we address the implications of this work in the context of implantable brain– machine interfaces for clinical applications and basic neuroscience. We present results illustrating the performance of our neural decoding architecture in an initial Results Section, which includes a subsection discussing techniques for noise reduction. In the Methods Section we describe the acquisition and format of our input signals, and develop the decoding and smoothing algorithms themselves. A Methods subsection describes in detail a concrete, hardware implementation of the neural decoding architecture in a low-power field-programmable gate array (FPGA).

## Results

### Neural Decoding

We applied our neural decoding system to decode head-position trajectories from place cell ensemble activity in the hippocampus of a behaving rat. Place cells in rat hippocampus exhibit receptive fields tuned to specific locations in the environment [48]–[50]. Our system was able to decode temporal firing patterns of ensembles of such cells in real-time simulations using recorded neural data. Spike train inputs were derived from spike-sorted tetrode recordings from the hippocampus of a rat bidirectionally traversing a maze for food reward, as described in [51]. In the example described here, the training phase consisted of a 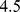-minute interval during which the rat traversed the entire maze once in each direction. This training interval directly preceded the 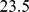-minute testing interval, during which the rat traversed the maze three times in each direction.

In the context of our place-cell– based position decoding problem, the states to be decoded, 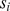, 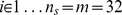, are 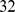 equally sized, discrete sections of a one-dimensional track maze, constituting an arbitrary discretization of the continuous, linear, 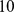-meter track. The track was unbranched but contained several right-angle turns.

**Figure 3:**
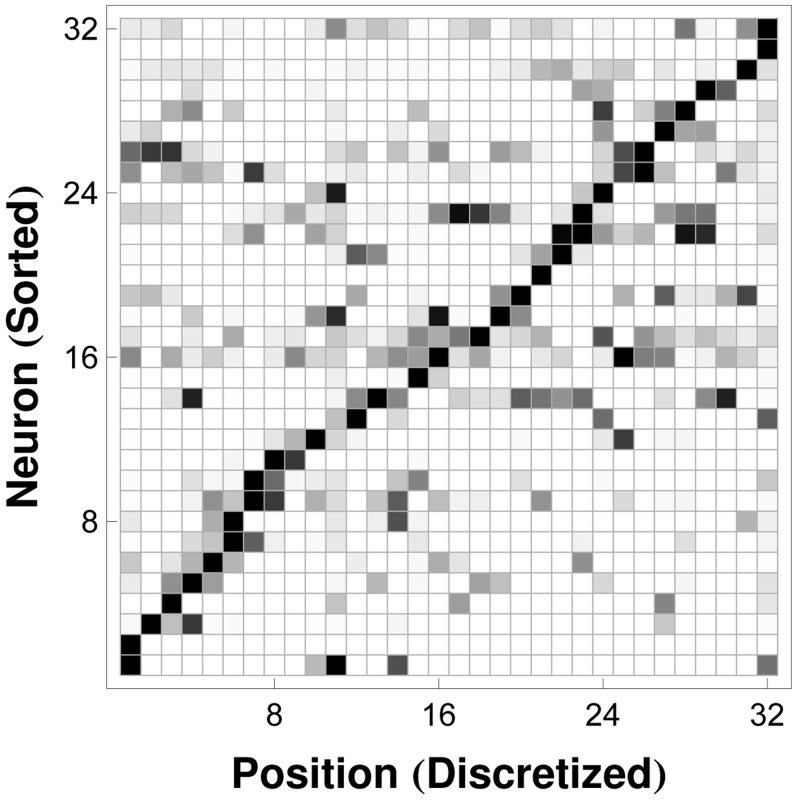
Encoding of Position by Place Cell Receptive Fields. Normalized spike rate for each of 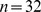 neurons in 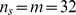 equal-length intervals along a one-dimensional track maze. Neurons (rows) have been sorted according to their positions of maximal activity to illustrate that the receptive fields of the place cells in this population cover the one-dimensional space of interest. Neuronal spike rates for each cell in each state (row elements) have been normalized to the highest spike rate (maximal row element) exhibited by the particular cell over all states. (Black: Maximal Spike Rate, White: Zero Spike Rate, Gray: Intermediate Spike Rates.).

Figure 3 illustrates the encoding of position in our ensemble of 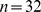 place cells. The columns of the color-coded array are normalized representations of spike activity for the place cells in the ensemble, with bins (columns) corresponding to discretized positions in the one-dimensional track maze. The rows have been sorted based on the locations associated with maximal spike activity. Figure 3 shows that the receptive fields within this ensemble of neurons are distributed over the available one-dimensional space, forming a basis for effective decoding.

**Figure 4:**
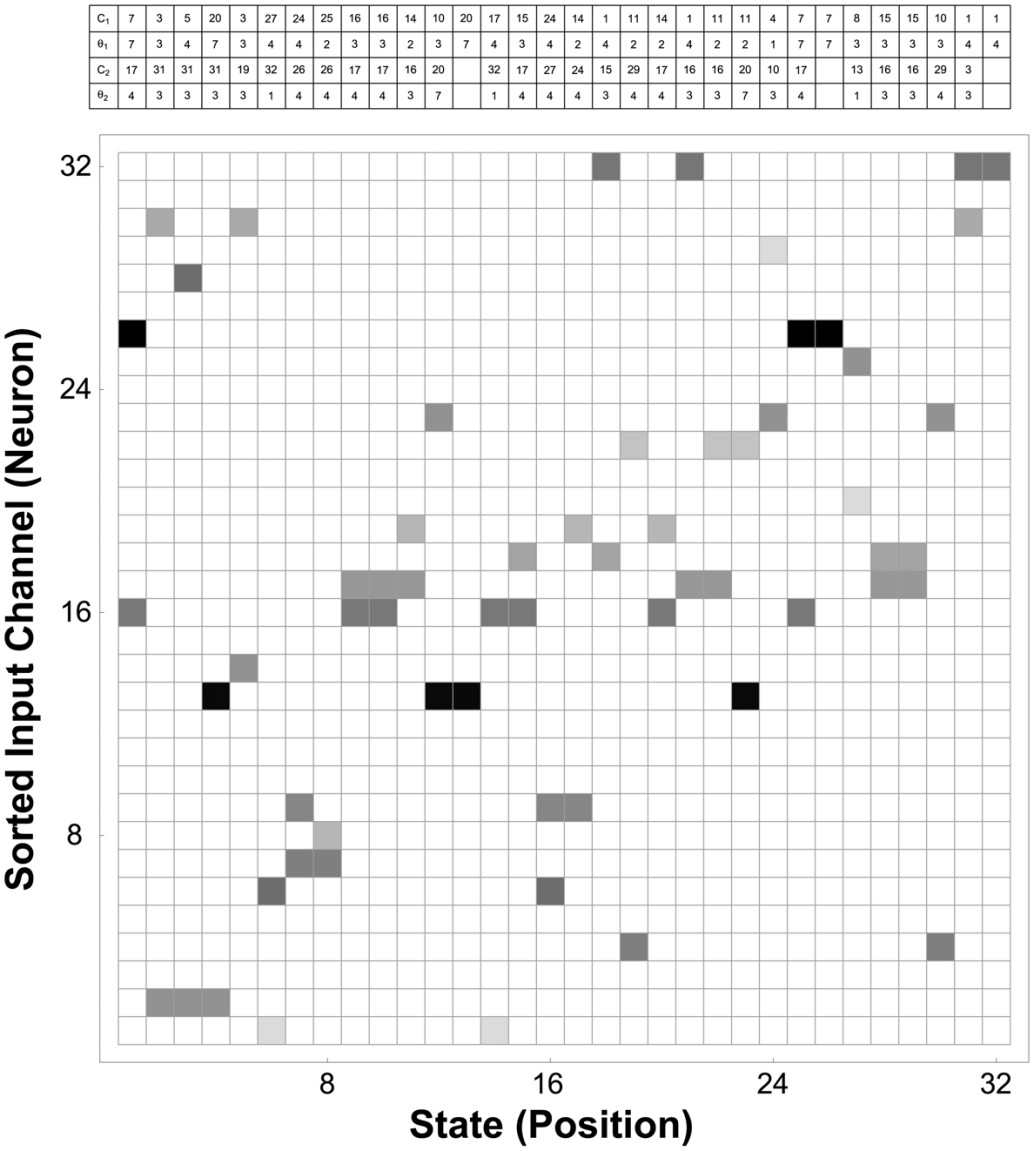
Decoder Logic Program: Finite-State Automaton Rules for Neural Decoding. Decoding template array 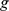 stored in system memory, resulting in the output shown in Figure 5, using the 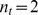 most informative threshold values for each position state. Some elements of the rule table (three pair) are empty, with corresponding columns having fewer than 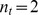 nonwhite elements, because the associated states had fewer than 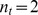 channels able to satisfy 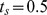 and 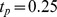, the minimum sensitivity and positive predictive value, respectively, for state decoding. (White: Unused, Light Gray: 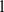 Spike per 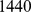-ms Window, Black: 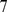 Spikes per 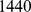-ms Window.) Intuitively, this set of templates can be understood as the tape-reading rules for a Turing machine, whose symbols are generated by the time-windowed spike counts on neural input channels, and whose states correspond to a discretized set of position states encoded by the underlying neuronal populations. At each time step, the neural decoder scans down each column in the array to determine the states, if any, whose rules have been satisfied; the decoded output elements 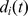 are set to 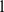 for those states, and to 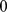 otherwise. The rules displayed graphically in the rectangular array are encoded numerically in the table displayed above the array (which is reproduced in Table 1). The columns of the table are aligned with the states in the array for which they contain decoding data, comprising the indices of the two most informative channels, 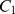 and 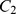, and the corresponding spike thresholds, 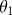 and 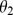.

Figure 4 graphically displays the data structure, 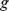, in which the decoding templates have been stored. We used 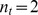, 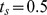, and 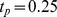 to compute the decoding templates, where 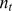 refers to the number of most informative channels used to decode each state, and 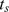 and 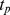 respectively denote the global minimum thresholds for the sensitivity and positive predictive value of state decoding, as described in the Methods Section. In Figure 4, 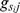 is displayed as an 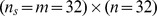 array, in which column 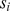 contains the template used to establish the spike count thresholds for state 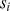. Hence, each column contains 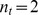 maximally informative elements, color-coded in one of 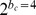 shades of gray, with dark values corresponding to a threshold spike count of 7 spikes counted within the integration window, light gray corresponding to a threshold spike count of 1 spike counted, and white corresponding to channels that are not used in the decoding of that state. The decoding rules displayed graphically and as fine print in Figure 4 are reproduced in Table 1.

**Table 1:** Tape-Reading Rules for a Turing Machine Decoder.

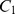	1	1	10	15	15	8	7	7	4	11	11	1	14	11	1	14	24	15	17	20	10	14	16	16	25	24	27	3	20	5	3	7
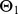	4	4	3	3	3	3	7	7	1	2	2	4	2	2	4	2	4	3	4	7	3	2	3	3	2	4	4	3	7	4	3	7
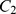		3	29	16	16	13		17	10	20	16	16	17	29	15	24	27	17	32		20	16	17	17	26	26	32	19	31	31	31	17
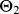		3	4	3	3	1		4	3	7	3	3	4	4	3	4	4	4	1		7	3	4	4	4	4	1	3	3	3	3	4

The decoding rules displayed graphically and as fine print in Figure 4 are reproduced here for clarity. The 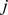
th column of the table corresponds to the 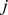
th position state (as reflected by the vertical alignment in Figure 4), and contains decoding instructions for the 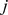
th state that are specified by the indices of the two most informative channels, 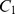 and 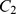, and their corresponding spike thresholds, 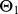 and 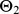. (As indicated in the context of Figure 4, some elements of the rule table are empty because the associated states had fewer than 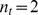 channels able to satisfy the minimum sensitivity and positive predictive value for state decoding.)

**Figure 5:**
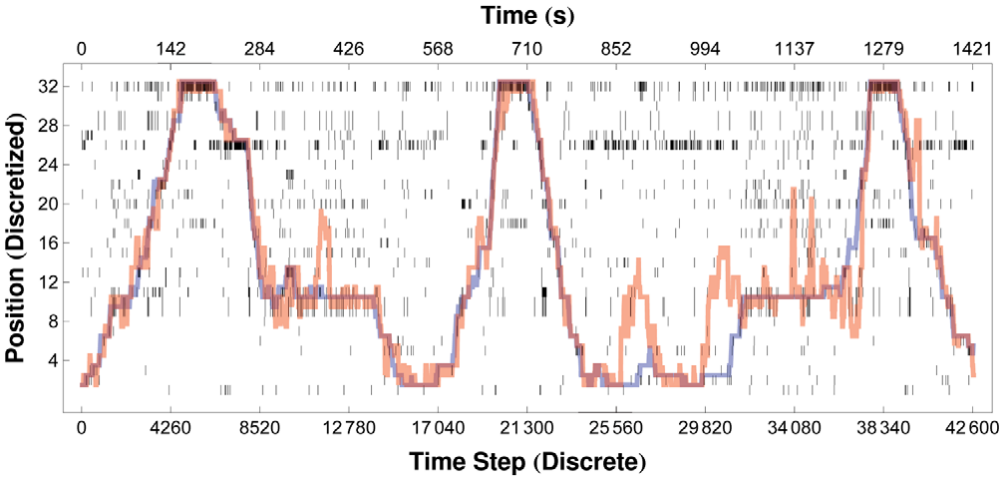
Decoder Output when Decoding Position from Hippocampal Place Cells. Our system decodes the location of a maze-roaming rat, from spike trains recorded from thirty-two hippocampal place cells. Raw output of the decoding algorithm, 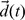, is shown as a raster array, with the output at each time step displayed as a vertical column of pixels (black pixels correspond to 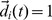, white pixels to 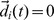). Red lines show the trajectories obtained after applying our Viterbi algorithm to the raw decoder output, as described in the text. The actual trajectories of the rat are shown in blue. Decoding accuracy and decoder noise are affected by the length of the time window over which spikes are collected at each time step: 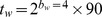 ms (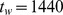 ms). Here the decoded trajectory matches the actual trajectory with a correlation coefficient of 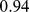.

Figure 5 displays the performance of our decoding algorithms at both the spike-to-state and state-to-state stages. At each time step, the output of the decoder is displayed vertically, with black pixels representing the ones in the binary vector 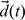. The locations of those ones are observed to cluster along a trajectory reflecting the position of the rat in time, with stray ones due to noise and decoder error. The effects of noise and spike-to-state decoder errors are reduced by the state-to-state smoothing algorithm (in this case a Viterbi algorithm implemented by the external unit), whose output is also shown. Using a window length of 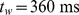, our decoded output matches the correct trajectory with a Pearson correlation coefficient of 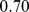; the correlation rises to 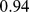 when the time window for input spike counting is widened to 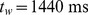, as spike rate estimation is more accurate and smooth in longer time windows, enabling better interstate discriminability. Our performance is comparable to those of other implementations [29], [52], [53].

### Compression Factor

Real-time decoding compresses neural data as it is acquired, by extracting only meaningful information of interest from a high-bandwidth, multichannel signal. For data acquired from an 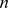-channel array and digitized to 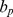 bits of precision at 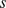 samples per second, our algorithm yields a compression factor of
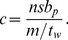
(1)In our work, data are digitized to at least 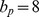 bits of precision, typically at a rate of 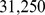 Hz; time windows are rarely shorter than 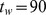 ms. In an 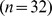-channel system decoding 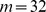 states, decoding therefore yields a compression factor of 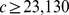. In this example we have used 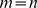 to generate a conservative value for 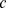; in practice, the number of states decoded is often fewer than the number of input channels, resulting in larger compression ratios.

### Computational Efficiency

We explicitly calculate the computational efficiency of our decoding architecture in the Methods Section, following a detailed description of its operation. We find that the total computational load, 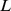, associated with neural decoding, scales as
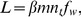
(2)where 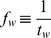, and 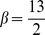 for the implementation employed here, as explained in detail in the Methods Section. Thus, for 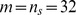, 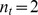, and 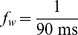, 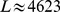 operations per second, or approximately 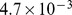 MIPS (millions of instructions per second).

### Noise Reduction and Cross-Validation

Input noise degrades the performance of the decoder, but our system has a number of mechanisms for mitigating the effects of noise. In considering the impact of noise on system performance, it is helpful to distinguish between correlated and uncorrelated noise, where the correlation is with reference to the collection of neural input signals, 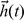, and 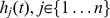, refers to the signal obtained from input channel 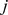. More precisely, the covariance matrix for 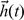, computed over a designated time interval, reflects the degree of correlation across input channels. In the context of neural signal recordings, cross-channel correlations (for electrodes spaced tens of micrometers apart) can in general arise from low-frequency components of the electroencephalogram (EEG) or from motion artifacts (movement of the recording array with respect to the brain, as may occur with head acceleration). Uncorrelated noise may be attributed to intrinsic properties of the recording system or to the biological signal itself, as discussed extensively in [9].

A useful feature of our internal decoding algorithm is its ability to explicitly suppress output noise and tune performance by adjusting 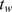, the duration of the window over which neural data are integrated at each time step before making a prediction. In particular, 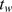 can be scaled in proportion to 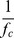, where 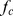 denotes a low-frequency cutoff in the noise spectrum. Lengthening 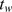 sacrifices system response speed for improved performance accuracy by integrating over more input data. Longer 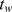 intervals require more memory to store decoding templates of correspondingly higher resolution, so the total memory available to the implanted system (in our case, 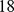 kbits of RAM) places an upper bound on decoding performance. As we illustrate in the ‘Neural Decoding’ subsection of the Results section, we are able to reduce the effects of output noise (effectively representing aggregated correlated noise that affects all input channels simultaneously) by lengthening 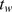. In particular, increasing 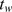 from 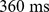 to 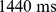 results in an improvement in the correlation of decoded with observed output from 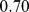 to 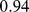.

The effects of correlated noise can also be suppressed in postprocessing, by the nonimplanted component of the system designated ‘External Computations’ in Figure 1. By virtue of its ability to use *a priori* constraints, such as those imposed by continuity as well as the physical properties and relationships of the states being decoded, the performance of the external system is not entirely limited by the statistical properties of the input signals and their associated noise levels.

Uncorrelated noise restricted to individual channels, associated with low channel-specific signal-to-noise ratios (SNRs), can be handled in the context of our decoding architecture by tuning the value of 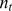 on a channel-by-channel basis. The system is robust to noise even in the absence of such channel-by-channel tuning, however. In order to assess the robustness the decoder to channel noise, we constructed a cross-validation analysis, in which we evaluated the performance of the decoder when the input consisted of 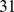 of the 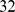 available channels of neural data, with the remaining channel replaced by noise. In this leave-one-out cross-validation scheme, we systematically replaced each of the 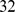 channels, one channel at a time, with a noise signal constructed to have the same spike rate as the original signal, but with spikes occurring at random times. Whereas the Pearson correlation between decoded (predicted) and experimentally observed animal position is 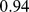 when all 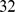 channels contain valid neural signals, the mean correlation falls to 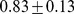 (maximum 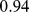) when only 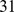 channels contain valid neural signals and one is replaced by noise in the fashion described. Thus, although system performance is somewhat degraded in the presence of this form of uncorrelated, single-channel noise, the overall performance, as measured by a mean correlation of 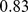 with observed animal position, nevertheless remains consistent with the levels of performance described elsewhere in the literature [29], [52], [53].

## Discussion

### Generalizations

Our system for neural decoding, implemented as described here, can be generalized in several ways.

First, consider the combinatorial logic we use to interpret spike train data. We designed the decoding architecture to facilitate applying primitive operations derived from the structure of neuronal receptive fields. We model the receptive field of a neuron with respect to a set of states as the probability distribution of action potential firing as a function of state index. Discretizing this model to a finite level of precision transforms the frequency distribution to a histogram over states. The general problem of evaluating the degree to which input spike counts represent evidence of particular states has often been cast in terms of Bayesian analysis [54]. In the context of a discrete-time digital implementation, however, the most general analysis of a set 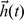 of input spike counts can be conducted by comparing each element 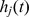 to a finite set of thresholds. As the set of all possible composite results of all such comparisons (made over all channels and all states) is enumerable and finite, discrete-time digital decoding can be implemented by a finite set of threshold-comparison rules. In particular, although the template-matching algorithm we describe here consists of logical conjunction operators applied to single, unidirectional threshold crossings on designated sets of channels, our decoding architecture is capable of using much more general combinational logic in analyzing 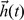. We note, further, that both the versatility of pattern matching [55] and the theoretical limitations of template matching [56] in the context of learning algorithms for computational neuroscience have been explored and reviewed in depth.

The combinational logic function we implement explicitly in this work, described by Equation 3, is appropriate when decoding from neuronal populations that encode information in receptive fields structured like those of the hippocampal place cells from which we demonstrate effective decoding here. The prototypical place cell has an approximately Gaussian, unimodal receptive field, which can be coarsely approximated by an impulse or threshold function that assumes a constant, nonzero value for states in which the cell is most active, and vanishes over all other states.

While such receptive-field encoding patterns are common, a more precise and more general model based on the one we describe here will have broader applicability. In a system with more memory or more logic circuits, a decoding scheme could use more logical functions with more conditions, operating on multiple upper- and lower-bound thresholds per channel. Such an approach could more smoothly model the state dependence of neuronal activity present in most receptive fields, through level-dependent conditions, where spike count levels are defined by pairs of upper- and lower-bound thresholds. This approach should be especially useful in decoding from multimodal receptive fields, or from input channels carrying unsorted multiunit activity, as in [57]. As indicated by the dashed ‘Operation Selection’ line in Figure 2, the most general decoder can employ different logical functions for each state.

Second, consider the information content of channel silence. Our decoding scheme takes action on the basis of observed spikes. Yet the absence of spikes also conveys information. A more general version of the system we describe here could exploit the information content of channel silence, constructing histograms and templates for time-windowed spike absence in analogy with those described here for observed spikes.

Third, consider cross-channel correlations in neural activity. The logical operations and probabilistic assumptions we have described here treat input channels, and indeed channel activity in each position, as independent. While it is convenient to assume such independence, neural activity across channels and positions may in general exhibit nonzero correlations. In a more elaborate version of our decoding system, such correlations could be exploited, for example by implementing combinational logic functions depending simultaneously on activity across multiple channels.

### Interpretations

Our decoding system, programmed as we have described here, illustrates how a simple universal computing architecture can implement effective, biomimetic neural decoding. In particular, the rule-based decoding program we describe can be understood as implementing a two-layer network of digitized integrate-and-fire neurons. The first layer of this network consists of 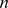 input neurons, corresponding to the 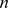 input channels of the system. The second layer consists of 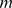 neurons, each connected to the 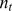 neurons in the first layer indicated by the decoding rules, and each synaptic weight set to the reciprocal of the corresponding threshold. Each second-layer neuron integrates its inputs for the duration of each time window, resetting to zero every 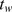, and firing when it accumulates a value of 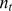. The output of the neurons in this second layer constitute the decoder output we have designated 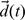.

The particular scheme we describe here for programming our decoding architecture has an intuitive interpretation as an emulation of a network of integrate-and-fire neurons. However, the rule-based programming structure of the system is extremely versatile, and by no means limited to intuitive, biomimetic computations. Indeed, the computational universality of such rule-based systems has been explored and described in detail by several investigators, including Wolfram [47].

## Methods

### Ethics Statement

This study uses behavioral and neurophysiologic data acquired from a live rodent. The data were provided to the authors by collaborators (T. J. Davidson) at the Picower Institute for Learning and Memory at the Massachusetts Institute of Technology. Experimental protocols were designed in accordance with guidelines set forth by the Department of Comparative Medicine at the Massachusetts Institute of Technology in the 2010 Laboratory Animal Users' Handbook, and were consistent with the goals of minimizing suffering in all work involving nonhuman vertebrate animals. All experimental methods were approved by the Committee on Animal Care at the Massachusetts Institute of Technology under protocol 0505-032-08, ‘Cortico-Hippocampal Ensemble Memory Formation.’

### Spike Detection

Our neural decoder is designed to process full-bandwidth, multichannel neural signals in real time, by interfacing directly with the digitized output stream from an array of amplifiers and neural recording electrodes. Such a direct connection of the logic to the amplifier array facilitates great flexibility in the spike detection and decoding algorithms that the associated system can implement. In the present work we describe a system for detecting neuronal action potentials (‘spikes’) using a single-threshold method; individual thresholds are programmable, and can be fine-tuned channel by channel. In the tests of the decoding system described here, however, we used acausally filtered, prerecorded neural data, as described in this section.

More advanced spike-detection methods are also possible. One such approach would be to implement dual-threshold detection, with or without a refractory period. Another would be to use more computationally intensive detection criteria, such as nonlinear energy operators [58]. However, we have found that a basic single-threshold method works well in practice.

Importantly, we can also estimate the noise level on each channel in real time. Estimating the noise level is necessary in high-channel-count systems because in such systems it is typically desirable to set spike detection thresholds automatically. A common approach is to set the detection threshold to a multiple of the noise level, isolating spike events with approximate statistical confidence bounds.

The results presented here were obtained from neural data collected as follows: Spike train inputs were derived from recordings from the hippocampus of a rat, while the animal repeatedly, bidirectionally traversed a linear track for food reward, as described in [51]. Spikes from multiple neurons were recorded on each of several four-microwire tetrodes, and the spikes were manually sorted (by identifying isolated clusters in spike amplitude space). Decoder input waveforms were then constructed by thresholding the spikes observed in isolated clusters, with the additional constraint that only cells with clear spatial modulation were included.

### Neural Decoding Algorithm

#### Overall Scheme of Operation

The objective in neural decoding is to infer an aggregate neural state, such as the intention to move a limb in a particular way, from signals sampled from a population of neurons encoding that state. The computational task of a neural decoding system operating in discrete time can therefore be described in the language of universal computing architectures, and our neural decoding architecture can be understood in terms of Turing-machine– like architectures with tapes and heads, as shown in Figure 1. At each time step, the system accumulates a sample of activity from a targeted neuronal population. This sample is interpreted as a symbol, or set of symbols, on the ‘tape’ read by our processor. The state of the system at that time step is inferred from the symbols by a discrete set of rules, stored in the memory of the system and used in reading the ‘tape.’

The rules for symbol interpretation are derived using a statistical procedure that we describe here in detail. Intuitively, the procedure treats neural spike counts from each input channel as test statistics, used to evaluate the probability that the system is in each of the possible states. The distributions of these test statistics, conditioned on being in or out of each of the possible states, are approximated as histograms collected during a learning phase. Maximally informative spike-count values (thresholds) can therefore be derived and converted to logical rules involving only comparison operations.

Our decoding architecture operates as follows:

Neural spikes are detected on each of 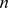 input channels using digitally programmable thresholds, as described in the Methods Section under ‘Spike Detection.’ Spikes detected from each channel during an observation window of duration 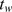 (typically tens to hundreds of milliseconds) are registered in 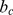-bit counters. At the end of each time window, the set of counter values is stored in an 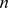-dimensional spike-count vector, 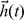, describing neural activity across all channels. This vector is compared, component by component, to a set of 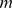 stored templates embodying the decoding rules. Each template 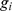, 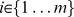 is also an 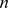-dimensional vector, and its components 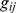, 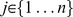 constitute spike-count thresholds for corresponding channels 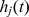 in 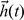. The data structure 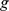 that contains the templates is therefore an 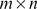 array, 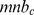 bits in size; one such template array is illustrated in Figure 4. The set of templates contains at least one member for each of the 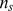 states to be decoded.

At the end of every time window, 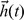 is compared component-wise to each of the 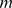 templates, 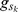, 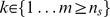, 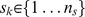; we index the templates in this way to make clear that we allow states to be encoded by multiple templates. The decoder output vector, 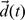, is then defined as follows:
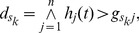
(3)where the condition specified in Equation 3 is the logical *and* of all the component-wise comparisons between the spike counts in the input at time 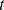, and the corresponding stored thresholds in the templates encoding a given state, 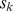. Thus, at every time step, the decoder independently evaluates the strength of the evidence that the state 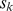 is encoded by the neural population over the time window 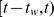. The decoder output, 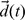, is an 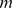-dimensional binary vector whose components coarsely encode the corresponding estimates as probable, 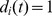, or improbable, 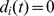.

In practice, as discussed in the Methods Section under ‘FPGA Implementation,’ the decoder output can be computed by implementing a reduced version of the full 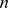-input logical *and*. This is accomplished by storing the templates 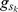 not as 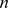-component vectors, but as 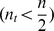-component vectors containing the 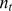 most informative threshold values for each state, paired with pointers to their corresponding input channels. This simplified version of the full decoding algorithm is useful in practice because it reduces the amount of memory and the amount of logic, and consequently the total power, required to implement the algorithm. The reduction is possible because in most practical applications the number of highly informative channels for any state is considerably smaller than the full number of channels in the system. The decoding algorithm itself permits selection of any value 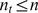, and the selection 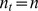 would correspond to the full decoding algorithm as reflected in Equation 3. In simplifying the implementation of the algorithm to save memory, logic, and power, a value of 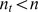 can be selected empirically to ensure that decoding performance remains sufficiently comparable to that achieved in the case of 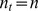. As we discuss in the Results Section under ‘Generalizations,’ and as indicated by the dashed ‘Operation Selection’ line in Figure 2, a more general form of the decoder architecture can also store a pointer to a logical operation to apply to the indicated channel thresholds. This pointer would select among alternatives to the operation described in Equation 3, which we apply uniformly across all channels. In a more general decoder, a more flexible scheme along these lines might be appropriate, in which optimized, channel-specific logical operators are selectively applied to each channel.

The decoding algorithm itself makes no *a priori* assumptions about the nature of the encoded states. As a result, it may be equally well suited to decoding tasks involving continuous trajectories (controlling a computer mouse, for example, or a prosthetic limb) and to those involving discrete and discontinuous decisions (such as typing on a computer keyboard, controlling the click-state of a mouse, or selecting from among preprogrammed grip states of a prosthetic hand). Decoder output predictions of multiple simultaneous states, null predictions, and state-to-state transitions that are implausible due to physical or other constraints, are all handled by the smoothing algorithm described in the Methods Section under ‘Trajectory Smoothing Algorithm.’

### Computational Efficiency

In order to determine the total computational load associated with our decoding architecture, we account for the number of operations required in each computational frame, defined as a single cycle through the input channels, in which a single spike-count vector, 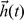, is acquired. Each spike-count vector must be compared against 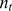 thresholds in each of 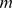 stored templates. As illustrated in Figure 2, each comparison is associated with the set of
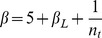
(4)operations enumerated in Table 2, where the parameter 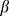 denotes the total number of basic operations per frame, and the parameter 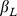 denotes the number of basic operations associated with the combinational logic used to compare incoming spike-count vectors to each stored template. In the implementation we demonstrate here, that logic consists of a single comparison, and so 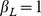. In general, however, when more intricate logic is used to evaluate 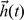, such as when a sum-of-products scheme is used to account for receptive fields with multimodal activity patterns (or, equivalently, input channels carrying multiunit spike activity), 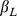 may be greater than 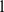.

**Table 2:** Basic Operations per Computational Frame of the Decoding Architecture.

Operation	Instances per Frame
Clock Counter 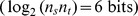	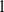
Memory Access	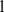
Multiplexer 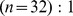	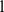
Comparison 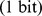	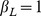
Binary Logic ( 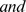 )	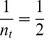
Shift Register 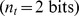	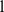
Shift Register 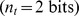	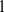
Total	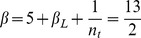

Detailed accounting of the basic operations required in each computational frame of neural decoding, corresponding to the system block diagram of Figure 2. The parameter 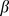 denotes the total number of basic operations per frame. The parameter 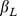 denotes the number of basic operations associated with the combinational logic used to compare incoming spike-count vectors to each stored template.

Spike-count vectors 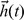 are acquired at frequency 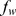, so the total computational load, as indicated in Equation 2, is 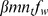.

### Computation of Templates

Decoding templates are constructed so as to be, in a statistical sense, both sensitive and specific to their associated states. By *sensitive* and *specific* we mean, respectively, that each template should detect its corresponding state with high probability when that state occurs, and that templates should discriminate well between states.

**Figure 6:**
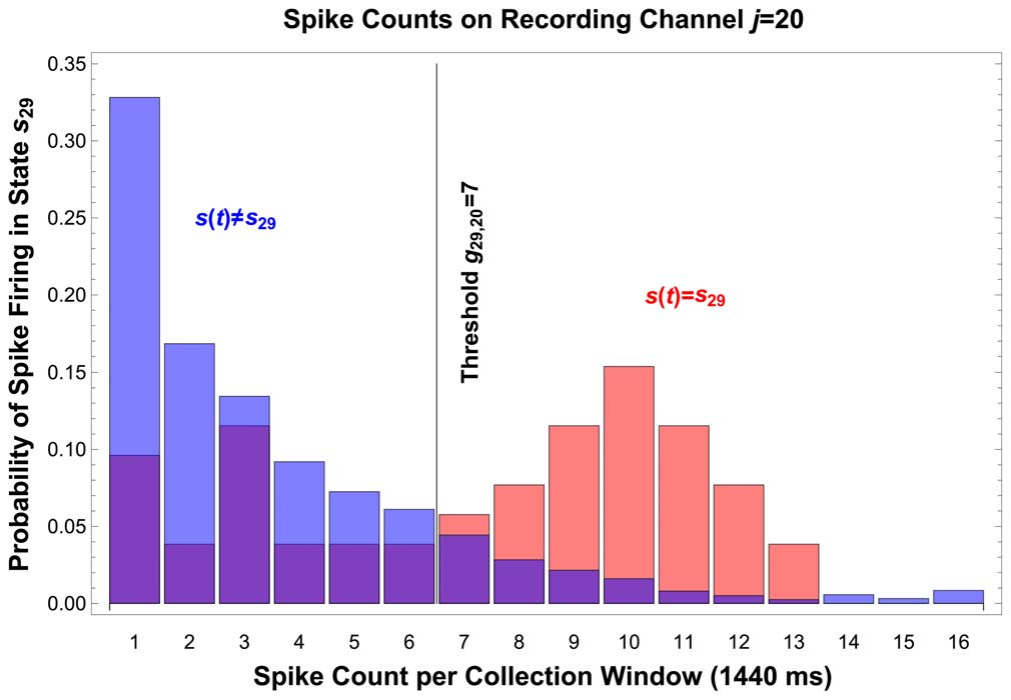
Histograms and Threshold. Histograms collected during the training phase of the decoding algorithm facilitate computation of thresholds for windowed spike activity, which are stored as templates in memory and used to discriminate between states. This histogram of spike activity, collected from recording channel 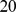, demonstrates that a threshold of 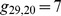 spikes per 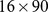-ms window, on recording channel 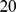, is sensitive and specific for state 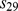 (Sensitivity: 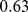, Specificity: 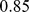, Positive Predictive Value: 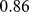). This threshold is written on the threshold tape used to program the internal unit of the decoder, and can be seen numerically in Figure 4 as the 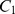 and 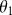 row entries of column 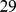, and graphically as the corresponding pixels in the rule array.

The decoding templates are computed based on data gathered during a training period, as shown in Figure 6, and are based on the approximation that the system state is stationary over the chosen time window, 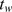. From the training data the decoder learns the tuning and firing rate properties of each input channel with respect to the defined set of states, in the following way:

The template construction algorithm begins by computing two sets, 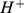 and 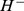, of joint frequency histograms for neural spike activity over all pairs of channels and all states. The sets 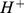 and 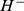 are organized by state and by channel. More precisely, 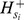, 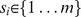, is a data structure containing 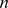 joint frequency histograms, 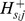, 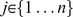, of the number of spikes detected on channel 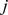 in a time interval of length 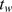 during which the system encodes state 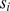. Similarly, 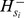 stores corresponding histograms of the number of spikes detected on channel 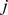 in a time interval of length 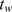 during which the system encodes a state *other than* 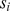. As described in the Methods Section under ‘Overall Scheme of Operation,’ a template 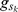 consist of a set of 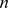 thresholds, corresponding to the minimum number of spikes that must be detected on each channel during a single time window in order to meet the criteria for state 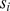.

We use the following heuristic for setting these thresholds, designed to yield decoding performance meeting at least a tunable minimum quality. We begin by setting two parameters, 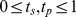, representing a global minimum sensitivity and a global minimum positive predictive value, respectively. We then consider each pair of histograms, 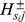, and for every possible threshold value from 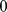 to 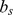 we compute the sensitivity and positive predictive value with which the threshold discriminates between 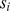 and 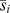, where the overbar denotes the set complement (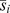 signifies *not*-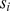, any state other than 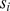). The template value 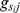 is set to the lowest threshold value yielding a discrimination sensitivity greater than or equal to 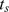 and simultaneously a positive predictive value greater than or equal to 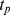. In the reduced implementations described in the Methods Section under ‘Overall Scheme of Operation,’ 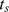 and 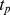 can be tuned either globally or on a state-by-state basis until no template contains more than 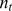 predictive thresholds.

### FPGA Implementation

Figure 2 shows our architecture for implementing the neural decoding algorithm described in the Methods Section under ‘Overall Scheme of Operation.’ As a practical demonstration of our computational architecture for neural decoding, we implemented our system in a low-power, 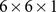-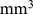, AGL060 FPGA of the Actel IGLOO Low-Power Flash family, which is tightly constrained by a number of parameters [59]. The measured power consumption of the decoding architecture, obtained as the difference between total power consumption of the FPGA (the product of operating current and the 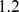-V supply voltage) when it is programmed to contain or exclude the neural decoding architecture, is 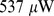. We note that the same architecture, implemented in an advanced-process application-specific integrated circuit (ASIC), should consume significantly less power, as even the lowest-power FPGAs are power-inefficient in comparison with custom ASICs. For example, custom digital architectures are often an order of magnitude more efficient than general-purpose architectures, such as those of FPGAs [9].

Our system accepts input from 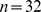 channels carrying digitized neural data from an array of recording electrodes and neural amplifiers [24]. The signal on each channel is thresholded to detect spikes, as described in the Methods Section under ‘Spike Detection,’ using a programmable comparator. Threshold-crossing events on each channel are registered by a 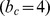-bit windowed counter, and the counters on all channels reset synchronously after every interval of 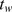. The set of counter values registered at the end of each time window constitute the input spike-count vector 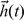 described in the Methods Section under ‘Overall Scheme of Operation.’

As described in the Methods Sections under ‘Overall Scheme of Operation’ and ‘Computation of Templates,’ the decoding algorithm functions by evaluating a set of 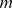 logical expressions at each time step. The elements of these expressions are drawn from 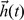 and the 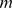 templates, 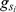; the templates thus implement programmable rules in combinational logic that are stored in the memory of the FPGA. In order to compress the decoding algorithm into the limited memory of our FPGA, we implement a reduced version of the template matching scheme, as described in the Methods Sections under ‘Overall Scheme of Operation,’ with 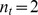 rules per state; the thresholds and corresponding pointers are stored in RAM.

In our implementation we use 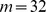 reduced templates to decode 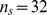 states. At each time step, for each template, the 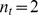 counters identified by the template pointers are compared to the corresponding thresholds stored in the template. The logical results of these comparisons are stored in 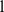-bit shift registers, and each component of the decoder output, 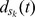, is formed from the logical *and* of the associated 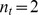 registers. The 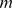 components of 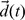 are saved in an 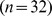-bit shift register, and 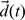 is transmitted every 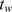, after all 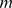 templates have been applied.

### Trajectory Smoothing Algorithm

Our decoding architecture separately implements routines based purely on *spike-to-state* correlation statistics, and routines based on physical or on other constraints on *state-to-state* transitions, independent of neuronal activity. This separation permits us to prioritize the former, the decoding computations most closely associated with raw neural activity and most critical for data compression, for execution within the implanted component of the system, and benefit from its inherent compression capabilities. The latter routines, described in this section, can then operate on the compressed data, outside the body. In the context of an implanted system with a small power budget, this scheme conserves power that would otherwise be spent in telemetry, by greatly reducing the bandwidth required for data transmission; and in computation, by offloading some of the decoding to the external components of the system. It also preserves algorithmic flexibility, as spike-to-state decoding, based on a generalized notion of neuronal receptive fields, is far less context-dependent than state-to-state decoding, which may employ a very broad range of constraints and context-specific statistical priors. For example, the priors employed to constrain letter-to-letter and word-to-word transitions in a typing task are very different from those used to smooth the trajectory of a prosthetic limb, yet both decoding problems can be addressed at the spike-to-state level by a pattern matching algorithm of the kind we describe here. The external unit, more accessible and less subject to power and size constraints, is better suited to tasks requiring more intense computation and frequent reprogramming.

We applied our decoding system to a problem involving the decoding of continuous movement, as described in the Results Section under ‘Neural Decoding,’ and so the state-to-state component of our decoding algorithm involves reconstruction of smooth trajectories from the spike-to-state predictions of the decoding algorithm described in the Methods Section under ‘Neural Decoding Algorithm’ and ‘FPGA Implementation.’

The classic Viterbi algorithm is an efficient, recursive approach to state sequence estimation; it is optimal in the sense that it generates a maximum *a posteriori* probability estimation of the sequence of hidden states in a finite-state, discrete-time Markov process operating in memoryless noise [60]. In casting our decoding problem in terms of a discrete-time, finite-state Markov process, we can construct trajectories by treating discretized positions as states in a hidden Markov model, and applying a modified Viterbi algorithm to convert the decoded output into a maximum-likelihood trajectory.

In the context of our decoder, the observed states are the values generated at the output of the decoder, 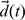, at each time step, 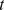. The actual positions 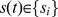 at the corresponding times are the associated hidden states. Implementing the Viterbi algorithm requires recursively computing
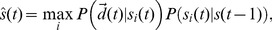
(5)where 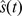 is the optimal estimate for the trajectory position at time step 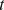. In the following paragraphs we explain the other terms in Equation 5, as well as our approaches to computing them.

The first term in Equation 5 denotes the probability of obtaining the decoder output 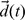 when the corresponding, true position is 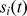. We use the following method to estimate these conditional probabilities. As the decoder output 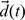 consists of 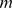 single-bit indicators reflecting the probability that 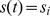, we treat each component 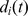 of 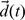 as being independent of the other 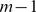. Under this assumption of independence,
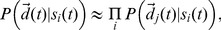
(6)where the index 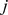 in Equation 6 runs over all components 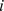 of 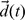 for which 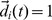. We estimate the probabilities forming the individual terms in the product of Equation 6 empirically, by computing a confusion matrix, 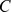, describing the performance of the decoder during its learning period. The element 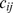 of this confusion matrix is the ratio of the number of time windows during which the decoder generated 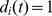 when the correct state was 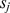, to the total number of time windows during which the decoder generated 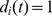; hence, 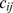 approximates 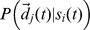.

The second term in Equation 5 represents a probability of transition from one state to the next; in our system, these transitions correspond to movements from one position to another. Such movements must obey physical constraints, and so the most probable transitions at adjacent time steps are those between each position and itself, and between a given position and its nearest neighbors. We therefore set the prior probability of transition between positions 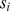 and 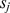 according to the diffusion-like expression in Equation 7:
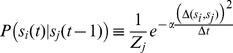
(7)

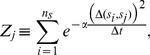
(8)where 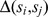 denotes the physical distance between the indicated states, and 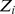 is the normalization constant for transitions from 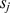 to 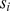. The inverse proportionality to 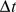, the time interval since the decoder generated at least one nonzero bit, permits transitions between more distant states with increased probability as time elapses, by broadening the probability distribution with elapsed time between informative observations; 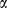 is a constant, related in our system to mean speed of movement, that tunes the rate of this spreading. We set 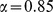 to obtain the results shown in Figure 5.
